# Novel COVID-19 vaccine hesitancy and acceptance, and associated factors, amongst medical students: a scoping review

**DOI:** 10.1080/10872981.2023.2175620

**Published:** 2023-02-14

**Authors:** Robyn Pandher, Justin L C Bilszta

**Affiliations:** Department of Medical Education, Melbourne Medical School, University of Melbourne, Parkville, Australia

**Keywords:** Medical student, COVID-19, vaccine hesitancy, vaccine acceptance, vaccine refusal

## Abstract

Medical students are likely to be exposed to COVID‐19 patients so achieving high vaccination coverage rates for this group of healthcare workers is important, as is their potential as vaccination role models. The aim of this scoping review was to evaluate the current literature to determine the rates of COVID-19 vaccine hesitancy and acceptance, and associated factors, amongst medical students. Systematic searches of the Medline Ovid, Embase, PubMed, and Education Resources Information Centre (ERIC) online databases was conducted for relevant articles with keywords: ‘COVID-19’, ‘vaccine hesitancy & acceptance’ and ‘medical students’. Articles were included for review if they reported the rates of vaccine hesitancy and acceptance, and associated factors, amongst medical students. Of the 258 articles identified, 52 met the inclusion criteria and underwent full-text review. Rates of vaccine hesitancy ranged from 5.4−86.7%, with generally positive attitudes towards COVID-19 vaccination. The main factors associated with vaccine hesitancy were concerns about the safety and efficacy of vaccines due to their accelerated development, being a pre-clinical medical student, and low perceived personal risk of COVID-19 infection. Inconsistencies were found for the influence of gender on attitudes towards vaccinations. Previous vaccination behaviours were predictive of willingness to receive the COVID-19 vaccine. Knowledge about COVID-19 vaccinations and their importance was found to be deficient amongst vaccine hesitant medical students. Generally, medical students express low levels of vaccine hesitancy. However, due to the variability in the factors associated with vaccine hesitancy across different populations and the dynamic and contextual nature of hesitancy, it is recommended that vaccination intent and associated attitudes are monitored on a longitudinal basis. It is important to map vaccine hesitancy at a local level to allow medical schools to develop strategies to encourage vaccination specific to their school’s needs.

## Introduction

Coronavirus disease (COVID-19) was declared a global pandemic on the 11th of March 2020 by the World Health Organization (WHO) [1]. Originating in Wuhan, China, the causative agent was identified as the SARS-CoV-2 virus in December 2019. As of 16 January 2023, there have been over 670 million confirmed cases globally and over 6.7 million deaths attributable to the infection [2].

The COVID-19 pandemic strained healthcare systems across the globe with shortages in essential healthcare resources – healthcare workers (HCWs), personal protective equipment (PPE), ventilators, and hospital beds [3]. There were significant HCW shortages due to deaths and COVID-19 related absences. As a result of increased patient burden and resource limitations, greater rates of burnout amongst the healthcare community were experienced. Shortages in PPE placed HCWs and patients at significant risk of exposure to infection [3].

The urgent development of a vaccine against COVID-19 was regarded as essential to controlling the spread of infection. Vaccination is one of the most important public health protection measures to decrease COVID-19 transmission and protect people from getting seriously ill, being hospitalized, and dying [4]. Multiple vaccines have been rapidly developed against the virus and shown to be efficacious and safe [5,6]. Nonetheless, acceptance of these vaccinations and the advent of vaccine hesitancy (VH) and vaccine refusal (VR) continues to be of concern.

Vaccine hesitancy is defined by the WHO as ‘… [a] *delay in acceptance or refusal of vaccination despite availability of vaccination services*’ [7]. It is listed as one of the top 10 threats to global health and poses direct and indirect threats to public health and wellbeing [7]. VH is a dynamic paradigm influenced by context, time, place, and vaccine availability. VH lies on a spectrum between individuals who accept all vaccines without question (i.e., vaccine acceptance, VA) to complete refusal to be vaccinated (i.e., VR) regardless of evidence to vaccine efficacy and safety. VH individuals represent those whose attitudes lie between VA and VR [8]. VH towards COVID-19 vaccines is an obstacle to immunization efforts and potentially hinders efforts to end the pandemic [9]. The effectiveness of any vaccination effort is dependent on the percentage of the population willing to get vaccinated; it has been estimated that at least 70% of the population must be vaccinated to end the current pandemic [10].

VH amongst HCWs is of particular concern as they are likely to encounter COVID-19 infected patients during clinical practice [11]. The causes of HCW VH are varied and include factors such as concerns around vaccine safety and efficacy and/or waiting for more information about safety and efficacy; concerns over the speed of vaccine development and release; mistrust of government and institutions; perceived infringement of personal liberties; and/or belief that vaccination is unnecessary [12,13]. Frontline HCWs were found to have three times the risk of reporting a positive COVID-19 test and predicted COVID-19 infection when compared with the general community, even after other risk factors were considered [11]. In response to workforce shortages, medical students, contributed to the pandemic response in a variety of clinical (i.e., frontline) and non-clinical capacities [14–16]. Medical students have the potential to infect vulnerable patients and contribute to the spread of infection as asymptomatic carriers [17,18]. As a result of this, many universities, health facilities, and medical schools across the globe, including Australia, mandated COVID-19 vaccination, including medical students, to engage and work in clinical settings [19].

Medical students, as future healthcare providers, are regarded as trusted sources of healthcare information by the public and play a critical role in influencing patients, relatives, and friends’ vaccine attitudes and intentions [20]. Effective communication by healthcare providers to patients about the benefits and risks of vaccines, their value, importance, and safety increases patient confidence in vaccination decisions [21]. Medical students in the clinical setting should be confident about the safety and effectiveness of COVID-19 vaccines and should be taught how to advocate and recommend for vaccination to their patients.

A preliminary search of the literature identified two articles which reviewed COVID-19 VH amongst medical students. Ulbrichtova et al. (2022) evaluated the prevalence of COVID-19 vaccination in medical students worldwide finding there was significant variation in the rates of VH across the globe based on a country's development status and the pandemic’s impact [22]. However, there was no analysis of factors associated with VH/VA. Similarly, Venkatesan et al. (2022) reported reasons for VH and motivating factors; however, there was little consideration of the influence of sociodemographic factors on vaccine intent [23].

Many cross-sectional studies have been conducted on the topic of COVID-19 VH in medical students; however, there are few systematic reviews on the topic which report on both the rates of VH/VA, reasons for hesitancy and associated sociodemographic factors.

Thus, the aim of this scoping review was to evaluate the rates of COVID-19 vaccine hesitancy and acceptance, and associated factors, amongst medical students. The specific research questions we sought to answer were as follows: i) what are the rates of VH, VA, and VR towards the COVID-19 vaccine among medical students?; ii) what are the predictors for hesitancy to vaccinate?; and iii) what factors – personal, sociodemographic, and cultural – influence COVID-19 vaccine uptake attitudes?

## Methods

This study adopted the ‘Preferred Reporting Items for Systematic reviews and Meta-Analysis extension for Scoping Reviews’ (PRISMA-ScR) reporting protocol to conduct the scoping review [24]. A copy of the PRISMA-ScR checklist is included in the Supplementary Materials. As per the recommendation on the PROSPERO website regarding scoping reviews (https://www.crd.york.ac.uk/prospero/), the review protocol was not registered.

### Search strategy

A comprehensive literature search using the electronic databases: Ovid Medline, ERIC, Embase, and PubMed for full-text articles reporting COVID-19 VH/VA and associated factors (see Supplementary Materials for example search strategy). The subject and text word search were performed separately in all databases and Boolean operators ‘OR’ or ‘AND’ were utilized to combine the terms. Hand searching of the reference lists of articles identified through the database search found additional papers which were included in the review.

### Inclusion and exclusion criteria

Only full-text articles were included. To be eligible, studies had to be primary cohort or cross-sectional in nature, reporting on medical students specifically and published in English. No publication date limits were placed on the search strategy. Studies were excluded if they were opinion pieces or review articles, and/or if they did not stratify the student population based on healthcare discipline (e.g., medical, nursing, dentistry, physiotherapy, and/or other allied health disciplines). Studies that did not report VH/VA rates were also excluded, as were articles that surveyed students’ attitudes towards vaccines other than the COVID-19 vaccine specifically.

### Data extraction and charting

References were uploaded into Covidence to allow sorting and review. A data extraction table developed in Excel was used to record the included article characteristics. The following information was extracted:

- *Article details*: first author and publication year.
- *Sample size*: number of students invited to participate and number that responded.
- *Population characteristics*: stratification of students by year of study, age, and gender.
- *Student rates of COVID-19 VH*: the rate of students reporting unwillingness to receive vaccination.
- *Student’s rates of COVID-19 VA*: the proportion of students reporting willingness to receive/have received vaccination.
- *Student’s rates of COVID-19 VR*: the proportion of students who reported outright refusal of vaccination.
- *Reasons for VH*: factors that contribute to vaccine hesitant attitudes in medical students.
- *General attitudes towards vaccination*
- *Prior vaccination uptake attitudes*
- *Social determinants associated with VH/VA*: age, gender, socio-economic status, cultural, and religious beliefs that contribute to vaccine attitudes.
- *Limitations*: identified study limitations.

### Synthesis of results

Thematic analysis was conducted to identify commonalities between the included studies. As a scoping review, a critical evaluation of the included literature was not performed and no determination as to the quality of the evidence/outcomes reported in each included study was made. No inferences were made about VH/VA rates, and associated factors, if they were not explicitly stated. Both authors (RP & JB) independently screened the titles and abstracts of the search results, and any discrepancies were resolved by discussion to reach consensus. Data extraction and charting results of included studies were undertaken by the first author (RP). The second author (JB) independently reviewed the data extraction and charting results once this process was completed by the first author.

## Results

### Database searches and article inclusion

From the primary search conducted between May and July 2022, 258 articles were identified for screening (18 from Medline, 155 from PubMed, 14 from CINAHL, 3 from Hand search & 68 from Embase). Eighty duplicate citations were excluded, leaving 178 articles for screening based on title and abstract. During screening, 81 irrelevant articles were excluded, resulting in 97 undergoing full-text review. Of these, 44 articles were excluded as they did not meet the inclusion criteria. A total of 53 articles were included in the final analysis. One article was excluded during the analysis process, resulting in 52 articles being reported in the final review (see Supplementary Material for the PRISMA diagram).

### Characteristics of included studies

A detailed description of the characteristics of the included studies is provided in Supplementary Material, Table A. They were conducted across 30 nations, with the greatest proportion from the United States (US) (*n* = 7, 13.5%), Poland (*n* = 5, 9.6%), India (*n* = 3, 5.8%), Saudi Arabia (*n* = 3, 5.8%), China (*n* = 3, 5.8%) & Pakistan (*n* = 3, 5.8%)

All studies were cross-sectional in nature, and online surveys were the most frequent method of data collection. Assessment of attitudes towards COVID-19 vaccination and the associated factors was evaluated using a variety of methods: online self-reported questionnaires [25–64], independent VH rating scales [65–71] or qualitative interviews [71]. Only five studies used the validated World Health Organization Strategic Advisory Group of Experts (WHO SAGE) working group vaccine hesitancy scale to define VH [72–76]. There was a significant methodological heterogeneity identified between the studies preventing quantitative analysis being conducted.

Of the 52 included studies, seven [30,50,66,69,73,74,76] did not report formal VH/VA rates. However, as these studies reported factors associated with intentions to vaccinate, the decision was made to include them for review.

### Rates of COVID-19 vaccine hesitancy, acceptance, and refusal

The percentage of VH among medical students in the studies reviewed ranged from 5.4% to 86.7% (see Table 1). Most studies reported VH/VA among medical students, with a small number [26,27,54,56,67] reporting absolute VR rates which ranged from 2% to 17.3%. Saied et al. (2021) reported the highest refusal rate with 17.3% of their medical student cohort refusing COVID-19 vaccination [56].

**Table 1.** Reported rates of VH, VA, and VR among medical students.

		Rates of		
Reference	Participants^β^ (*n*)	VH [*n* (%)]	VA [*n* (%)]	VR [*n* (%)]
*Altulaihi et al. (2021)* [25]	204	69 (34)	135 (66) vaccinated	NR
*Balan et al. (2021) [26]*	1146	64 (5.6)	1053 (91.9)	29 (2.5)
*Bolatov et al. (2021) [65]*	888	671 (75.6)	217 (24.4)	NR
*Chaves et al. (2021) [27]*	250	35 (14)	210 (84)	NR
*Cuschieri et al. (2021) [28]*	NR^¥^	NR (13.4)	NR (86.6)	NR
*Gala et al. (2022) [29]*	370	45 (19.6)	184 (80.4)	NR
*Gao et al. (2022) [31]*	652	102 (15.6)	550 (84.3)	NR
*Gautier et al. (2022) [32]*	348	56 (16.1)	292 (83.9)	NR
*Grochowska et al. (2021) [33]*	239	70 (29.3)	169 (70.7)	NR
*Habib et al. (2022) [34]*	1445	481 (33.3)	964 (66.7)	NR
*Holzmann-Littig et al. (2021) [75]*	1313	88 (6.7)	1225 (93.3)	NR
*Hosek et al. (2022) [35]*	358	35 (35)	323 (60.4)	NR
*Jain et al. (2021) [36]*	1068	113 (10.6)	689 (64.5) vaccinated 266 (24.9) yet to receive vaccine	NR
*Kanyike et al. (2021) [37]*	488	307 (62.9)	181 (37.1)	NR
*Katz et al. (2022) [38]*	104	9 (18.7)	95 (91.3)	NR
*Kausar et al. (2021) [39]*	356	59 (16.57)	297 (83.43)	NR
*Kaya et al. (2021) [67]*	739	190 (25.7)	44 (60.1)	105 (14.2)
*Kelekar et al. (2021) [40]*	167	38 (23)	139 (77)	NR
*Latif et al. (2022) [41]*	522	47 (9) not vaccinated	475 (91) vaccinated	NR
*LeAn et al. (2021) [68]*	392	91 (46.4)	301 (45.7)	NR
*Li et al. (2022)†* [71]	238	7 (4.7)	219 (92)	NR
*Lindner-Pawlowicz et al. (2021) [42]*	350	81 (23.1)	269 (76.9)	NR
*LoMoro et al. (2022) [43]*	929	60 (6.7)	869 (93.5)	NR
*Lucia et al. (2021) [44]*	167	37 (22)	126 (75)	NR
*Mahdi et al. (2021) [45]*	810	702 (86.7)	108 (13.3)	NR
*Mayan et al. (2021) [46]*	1899	127 (7.7)	1772 (93.3)	NR
*Mose et al. (2022) [47]*	122	49 (40.2)	73 (59.8)	NR
*Mubarak et al. (2022) [48]*	332	64 (16.3)	278 (83.7)	NR
*Mustapha et al. (2021) [72]*	166	83 (50)	83 (50)	NR
*Nguyen et al. (2021) [49]*	219	41 (18.7)	178 (81.3)	NR
*Oduwole et al. (2021) [51]*	200	18 (9)	182 (91)	NR
*Pastorino et al. (2021) [52]*	274	20 (7.3)	254 (92.7)	NR
*Peterson et al. (2021) [53]*	234	19 (18.2) unvaccinated	215 (91.9) vaccinated	NR
*Petravic et al. (2021) [54]*	311	16 (5)	255 (82)	40 (13)
*Raja et al. (2022) [64]*	217	96 (44.2)	121 (55.8)	NR
*Rosental et al. (2021) [55]*	321	38 (11.9%)	283 (88.1%)	NR
*Saied et al. (2021) [56]*	727	340 (46.8)	261 (35.9)	126 (17.3)
*Serbezova et al. (2021) [57]*	400	234 (4) not vaccinated	166 (35)	NR
*Shah et al. (2021) [58]*	274	81 (29.6)	193 (70.4)	NR
*Sovicova et al. (2021) [59]*	1240	348 (28.1) not vaccinated	892 (71.9)	NR
*Sugawara et al. (2021) [70]*	496	46 (9.3)	450 (90.7)	NR
*Szmyd et al. (2021) [60]*	687	55 (8.0)	632 (92)	NR
*Talarek et al. (2021) [61]*	411	22 (5.4)	389 (94.6)	NR
*Thekrallah et al. (2022) [62]*	283	48 (17)	235 (83)	NR
*Usman et al. (2021) [63]*	410	110 (26.7)	300 (73.3)	NR

VH, vaccine hesitancy; VA, vaccine acceptance; VR, vaccine refusal; NR, not reported.

^β^In studies with mixed cohorts, medical student cohort data only has been reported.

^¥^Total student numbers (*n* = 679) not stratified by health discipline. Rates of VH and VA were stratified by health discipline but only percentages reported.

^†^Results do not add up to 100% due to missing data.

### Factors associated with vaccine hesitancy and acceptance

Increased VH associated with concerns regarding the safety and efficacy of the vaccine, particularly the long-term adverse effects, were reported in 26/52 (50%) of the included studies. This was primarily related to concerns regarding the rapid development of the vaccine and lack of time spent in clinical trials (*n* = 14, 27%), with students wanting to wait and see how the vaccine affected people before getting vaccinated in 6 studies [35,36,53,64,65,76].

Low perceived personal risk of contracting COVID-19 infection, low vulnerability to severe infection, and belief in natural immunity [25,36,38,39,43,52,53,55,58,64,66] were also found to be associated with VH. Previous history of COVID-19 infection was shown to increase vaccine acceptability in some studies [45,64,67]; however, it was also shown to decrease vaccine acceptance [39,69].

There was a significant variation in the influence of gender on vaccination intent, with reports of *both* male [29,46,67] and female [37,43,45,54,65] gender being associated with greater VH. The influence of student age was inconsistency reported; some authors demonstrated younger students [25] were more likely to be VH but others [39] showed no association. Sovicova et al. found that medical students living at home with their families were less likely to be COVID-19 vaccinated compared to respondents who lived on the campus or on their own in a private residence [59]. This finding was not replicated by others. Both Sovicova et al. and Gao et al. found that medical students living in urban areas were less likely to be COVID-19 vaccinated compared to students living in rural areas [31,59].

COVID-19 vaccine types available to, or received by, medical students were not consistently reported, and no study evaluated if an association existed between vaccine type and VH/VA rates. Of those studies (*n* = 20/52, 38.5%) studies that did include a description of vaccine type, the breakdown was as follows: mRNA vaccine (*n* = 17/20, 85%), spike protein (*n* = 12/20, 60%), viral vector (*n* = 8/20’ 40%), and inactive whole viron (*n* = 6/20, 30%). It was also not clear from the included studies if the presence of Government COVID-19 vaccination mandates for medical students was associated with attitudes to vaccination. This was because not all included studies reported if mandates existed at the time of data collection, and no study evaluated if an association existed.

Prior vaccination attitudes were found to influence the intention to receive the COVID-19 vaccine; students with a history of adverse outcomes from another vaccine or had previously delayed vaccination due to a reason other than allergy or illness were found to have higher rates of COVID-19 vaccine hesitancy [43,53,59,60,65,76]. Talarek et al. (2021) reported 98.7% of the students who received the influenza vaccine in previous years were willing to receive the COVID-19 vaccine once it was made available [61]. Pastorino et al. (2021) also reported that influenza status was predictive of willingness to receive COVID-19 vaccination [52].

For information on vaccines and trusted sources when it came to making a vaccine decision, the main source of information was social media/internet [36,37,45,64] in the VH group. In contrast, trust in official information sources such as public health and governmental resources [39,40,54,65] was associated with greater willingness to get vaccinated.

The level of knowledge about COVID-19 and vaccination was assessed objectively in a small number of studies [34,46,58,67]. Students that were less knowledgeable and those that reported they needed more vaccine information reported higher rates of VH [28,31,38,39,44,58,59] compared to students more knowledgeable and confident in information received about the COVID-19 vaccine [31,46,67,69,74]. Lucia et al. (2021), the first study conducted in the US prior to vaccine introduction, reported 100% of the medical students in the VH group required more information before making vaccination decisions [44]. Similar findings were reported in Kelekar et al. (2021) with 100% of VH medical students reporting the same [40].

Many studies reported that medical students in their pre-clinical training years expressed greater VH than clinical students [29,31,34,36,39,46,59,61,65,67]. In pre-clinical medical students, VH rates ranged from 58.3% to 90% compared to 10% to 41.7% in clinical students.

## Discussion

VH continues to be a significant public health threat, more so during the COVID-19 pandemic [77]. The aim of this scoping review was to report the rates of VH and VA towards COVID-19 vaccines, and associated factors, amongst medical students. The following discussion combines the key findings and addresses the factors associated with VH/VA to enable the development of strategies and educational training programs targeted towards addressing these factors.

Overall, most medical students in the reviewed studies believed vaccinations are an important public health measure to protect themselves, the patients they encounter, and the general community from COVID-19 infection and severe disease [34,37,40,51,58,59,64,67]. However, due to the rapid development and introduction of COVID-19 vaccines many medical students planned to ‘wait and see’ before receiving vaccination. Concerns regarding the serious side effects of COVID-19 predicted intent to vaccinate in half of the reviewed studies. Kelekar et al. (2021) reported 89.2% of VH medical students expressing these concerns as a barrier to vaccination [40]. Supporting these findings, Cascini et al. (2022) found that amongst members of the general public concerns, COVID-19 vaccines related to their fast rate of development and regulatory approval and minimal efficacy and side effect safety data availability [78].

Generally, within the cohorts studied, rates of VH were low; however, the reported rates varied considerably. Our analysis suggests these inconsistencies may be based on temporal factors with higher rates of VH reported in studies conducted earlier in the pandemic. For example, Lucia et al. (2021), a study conducted in the USA preceding the approval of a COVID-19 vaccine for mass public vaccination, reported 23% of the medical students expressed VH, with the main concerns cited being the serious vaccine side effects, lack of information about the vaccination and lack of trust in the information received from public health experts [44]. Conversely, Mayan et al. (2021) a study conducted in the US following the introduction of public vaccination reported that a lower VH rate of 6.68% among their medical student population [46].

As the pandemic progressed, there was greater incentive to receive vaccination due to the introduction of compulsory vaccination requirements of selected population groups including HCWs and those at-risk, in several countries [19,79]. There were increasing requirements to get vaccinated to engage in education and resume social activities. Despite the increase in the number of individuals in the community receiving vaccinations, confidence in COVID-19 vaccines has dropped over time and there has not been a clear downward trajectory to the rates of VH [80,81]. Surges in VH have occurred across the timeline of the pandemic, particularly in relation to the development of new vaccine policies and mandates, new reports of vaccine risks, and spikes in COVID-19 infection in the community [82]. All included studies were cross-sectional in nature, meaning there are no longitudinal studies assessing changes in medical students’ perceptions and intents as the pandemic progressed.

Higher rates of VH were observed in African and Middle Eastern countries, with medical students reporting higher rates of belief in conspiracy theories about COVID-19 vaccines [34,48,50,63]. There was also greater mistrust in government and public health experts’ COVID-19 vaccine information and a higher level of trust placed in the opinions of family members, relatives, and social media when making vaccination decisions within VH populations [27,36,37,43,64,65]. It has been demonstrated this mistrust, which has been demonstrated in all HCWs and not just medical students, stems in part from perceived dishonesty of pharmaceutical companies and Government institutions involved in the production, distribution, and/or promotion of vaccines and vaccination protocols [12]. This belief may also be influenced by concerns over commercial profiteering as well as perceived pressured to administer vaccines and vaccine boosters despite the lack of safety and efficacy data. Others point to the development of mistrust because of HCWs believing that healthcare systems have failed to support them during a time of crisis [33] coupled with a perceived mishandling of the public health response to the pandemic [83].

Risk perception is a significant predictor of protection intentions and engagement with preventative health behaviors such as vaccination [84]. The perceived personal risk of contracting COVID-19 infection and the vulnerability to serious infection was associated with lower rates of VH [25,36,38,39,43,52,53,58,64,66]. There was division in terms of the acceptability of COVID-19 vaccine in medical students who had a history of COVID-19 infection, with some reporting that medical students were more willing to take COVID-19 vaccination after infection [45,64,67], whilst others reported the opposite [39,69]. The relationship between the higher level of VH in populations and the low perceived vulnerability to COVID-19 infection may be the result of vaccine complacency. Vaccine complacency occurs either when the perceived personal risk of the vaccine-preventable disease is low, the risk of vaccine is deemed to be greater than the infection or vaccines are not seen as a necessary preventative action [85].

Information gaps can promote complacency in the population with several studies reporting that students who did not feel confident in their knowledge of the COVID-19 vaccine and requiring more information before making a vaccine decision were more VH. Higher levels of knowledge of the vaccine and greater self-management skills were associated with greater willingness to receive the vaccine [31,46,58,67,69,74]. Shah et al. (2021) assessed medical students’ knowledge about COVID-19 vaccination using an objective knowledge questionnaire focused on questions about COVID-19 vaccinations’ mechanism of action and purpose [58]. They reported that those students with lower knowledge of COVID-19 vaccination expressed higher rates of VH.

Many medical students that expressed VH attitudes understood their responsibility as future physicians to be vaccinated and promote vaccination to patients and the general community [34,36,51,59]. Generally, positive attitudes towards other vaccinations and a history of receiving all recommended vaccinations was a motivating factor to receive COVID-19 vaccination [60,65,76]. Conversely, a history of experiencing adverse effects from a vaccination and delaying general vaccines for reasons other than allergy or illness was found to be associated with greater unwillingness to receive COVID-19 vaccination [43,53,59]. The influence that prior vaccine attitudes and experiences have on COVID-19 VH warrants further investigation.

Differences were noted between pre-clinical and clinical students’ intentions to receive the vaccine. Clinical students expressed lower rates of VH than their pre-clinical colleagues [29,31,34,36,39,45,46,59,61,65,67] this difference in attitudes might be reflective of the influence clinical exposure to COVID-19 patients and infectious diseases, levels of knowledge related to the vaccine, encouragement by clinical sites and educators to get vaccinated, as well as the development of skills in evidence-based medicine within the clinical student group.

The most common factors associated with VH are complacency, convenience, and confidence, referred to as the ‘3C’ model of vaccine hesitancy [8]. The data presented in this review suggest a proportion of medical students experience low *confidence* in the effectiveness and safety of vaccines and wished to adopt a ‘wait and see’ approach until such data became available. Medical students also experience a degree of *complacency* related to their perceived personal risk of contracting COVID-19 infection and vulnerability to serious infection. *Convenience* is less of a factor given medical students' position in the healthcare system, requirements to be vaccinated to continue their medical studies, and opportunities to access vaccination services relative to the public at large.

### Limitations

There are several limitations to our study. Articles may have been omitted due to the search strategy, inclusion criteria, and limitations on the English language. Exclusion of non-English language papers may have altered our findings as attitudes towards COVID-19 vaccine and the factors associated with them may be different in non-English-speaking countries. A critical evaluation of the included literature was not performed and no determination as to the quality of the evidence/outcomes reported in each included study has been made. The review protocol was not registered prior to commencement of the search strategy. There were 28 studies excluded from review as they did not stratify VH/VA rates and attitudes towards vaccination for medical students from the healthcare student population recruited. When evaluating students’ knowledge of COVID-19 vaccination, a limitation was identified in the ways in which the knowledge was assessed. Most studies used self-reported questionnaires as opposed to objective knowledge measures which have the potential to over-estimate students’ level of knowledge. Similarly, when evaluating students’ levels of COVID-19 VH/VA there was a lack of consistency in the methods used to collect data. As a result of this heterogeneity, generalizations relating to medical student’s vaccine intentions and the information obtained from each study are only relevant to the point in time and the student population being studied.

### Future directions

The influence of vaccine type on vaccine intentions is a topic for further research. The novel mRNA-based technology used in the most common vaccinations – Pfizer and Moderna – might have further contributed to VH sentiments due to the novelty of this technology. As noted, no study evaluated whether an association existed between VH/VA and the vaccine type. Similarly, differences in medical curricula, training, and educational environments could also be a contributing factor to the geographical and spatial variability in the vaccination intent of medical students. As the rates of VH/VA and associated factors are dynamic and fluctuate, it is recommended that future studies consider addressing this variability by exploring, in the same population, at different time-points of the pandemic. An additional factor to consider is whether VH/VA rates differ between medical students and students from other healthcare disciplines. This review only considered data reporting on medical students, although several studies included data from nursing and allied health students. It is important to determine if VH/VA rates differ between medical and other healthcare students and if so, the reasons why. This work could provide important insights into how educational curricula and environments, as well as profession culture, influence attitudes to vaccination. This could also highlight strategies which increase vaccination rates in healthcare students.

### Recommendations

Due to the variability in rates of VH/VA and associated factors based on the geographical and temporal factors, it is imperative that universities and educational institutions conduct surveys of their own student populations. Ideally, assessing the level of knowledge, VH/VA rates and associated factors amongst the student population should be undertaken using validated VH scales and objective knowledge-based questionnaires. Furthermore, given the dynamic and contextual nature of VH, it is recommended that vaccination intent is monitored on a longitudinal basis. Conducting population-specific studies will determine the need for introduction of targeted vaccine education strategies and allow administrators and educators to develop cohort-specific training programs to address the unique combination of factors and beliefs that promote VH.

## Conclusion

Overall, the general attitudes of medical students towards COVID-19 vaccination were positive. Significant influencing factors identified were concerns about safety and efficacy because of the accelerated development of COVID-19 vaccines, whether medical students were from a pre-clinical or clinical cohort, and general attitudes towards all vaccinations. It is important medical students possess positive attitudes towards vaccination and promote these attitudes to their patient population and the wider community. Due to the variability in the factors associated with VH/VA across different populations, it is important to map VH/VA at a local level and address concerns with interventions focused on the individual and community level. Effective tools to address VH include education and training programs, and the involvement of medical students directly into practice in the early years of their studies. Addressing COVID-19 VH amongst medical students will have a positive impact on the battle against the COVID-19 pandemic and future pandemics.

## Supplementary Material

Supplemental Material

## Data Availability

The data extraction sheet used and/or analysed during the current study is available from the corresponding author on request. Data are provided in the tables and appendices, and all data are based on published studies.
