# Supplementary material for: Novel COVID-19 vaccine hesitancy and acceptance, and associated factors, amongst medical students: a scoping review
Source: Med Educ Online. 2023 Feb 14;28(1):2175620. doi: 10.1080/10872981.2023.2175620 (PMC9930839; doi:10.1080/10872981.2023.2175620)
Supplement: Supplemental Material [file ZMEO_A_2175620_SM2120.zip › Supplementary files/Supplementary Material Captions.docx]

Supplementary Material Captions

*Figure 1: PRISMA-ScR checklist*

*Figure 2: Example search strategy*

*Figure 3: PRISMA diagram*
