# Supplementary figures and images for: Novel COVID-19 vaccine hesitancy and acceptance, and associated factors, amongst medical students: a scoping review
Source: Med Educ Online. 2023 Feb 14;28(1):2175620. doi: 10.1080/10872981.2023.2175620 (PMC9930839; doi:10.1080/10872981.2023.2175620)

**Supplementary Material. Search Strategy example from EMBASE search**


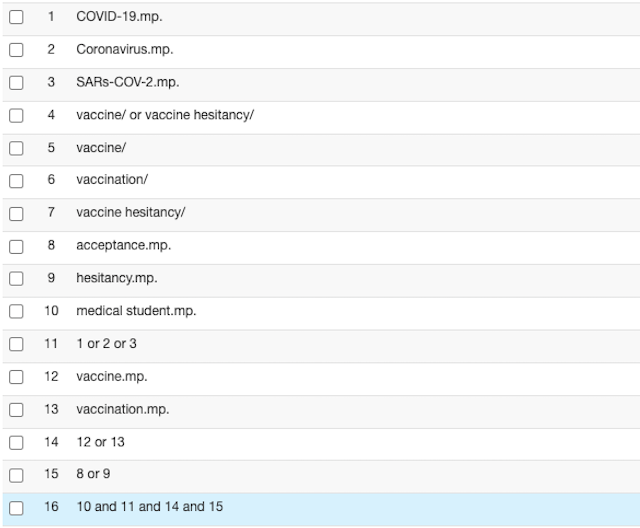

Supplement: Supplemental Material [file ZMEO_A_2175620_SM2120.zip › Supplementary files/Supplementary Material_Figure 2_Search stratedgy.docx]

**Supplementary Material. PRISMA Diagram**


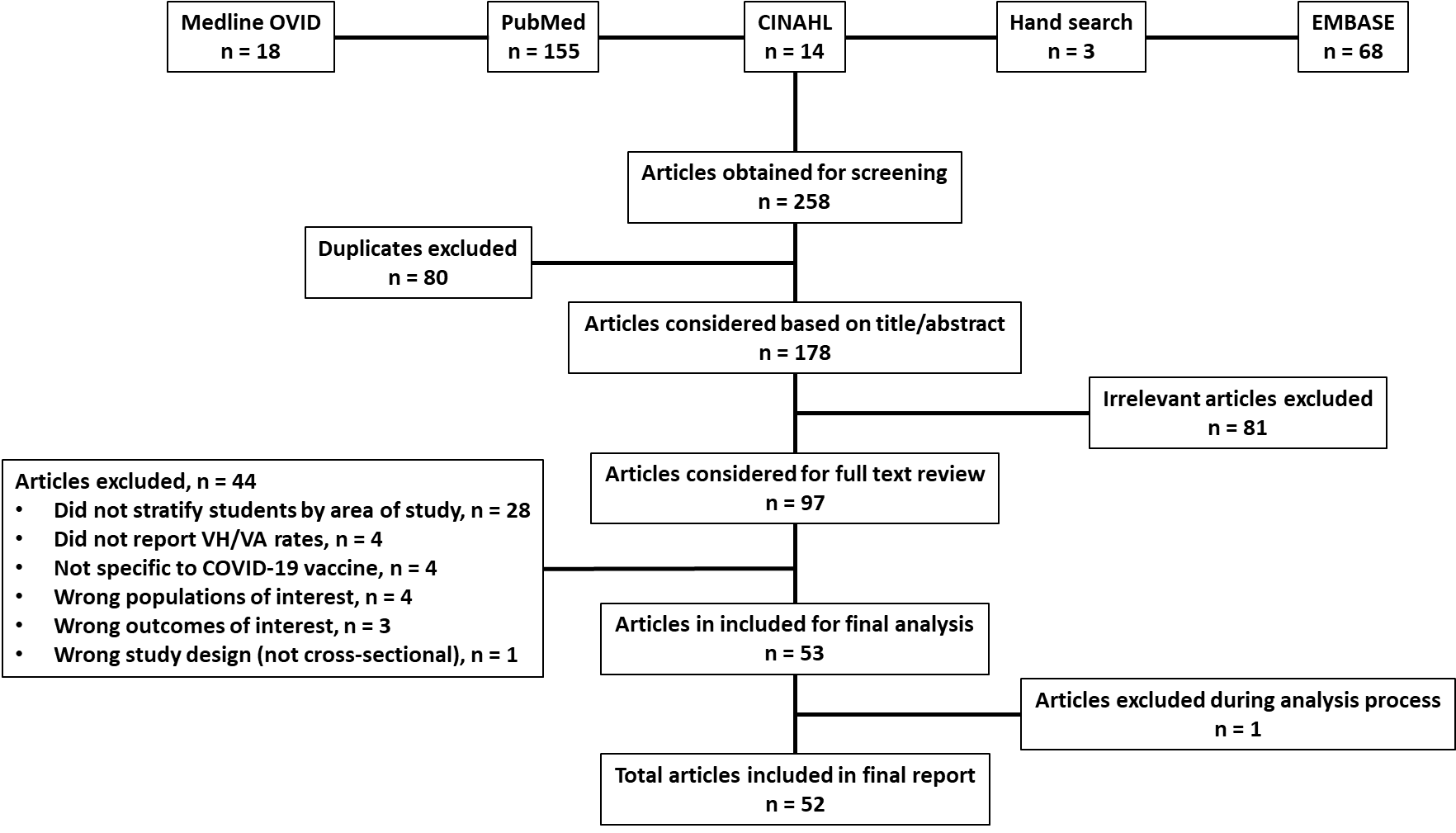

Supplement: Supplemental Material [file ZMEO_A_2175620_SM2120.zip › Supplementary files/Supplementary Material_Figure 3_PRISMA diagram.docx]
